# Adherence to safety barriers in medication administration: patients’ perception*

**DOI:** 10.1590/1518-8345.5383.3497

**Published:** 2021-11-08

**Authors:** Juliana Machado Campos Fleck, Rosana Aparecida Pereira, Ana Elisa Bauer de Camargo Silva, Fernanda Raphael Escobar Gimenes

**Affiliations:** 1 Universidade de Franca, Faculdade de Enfermagem, Franca, SP, Brasil.; 2 Universidade de São Paulo, Escola de Enfermagem de Ribeirão Preto, PAHO/WHO Collaborating Centre for Nursing Research Development, Ribeirão Preto, SP, Brazil.; 3 Universidade Federal de Goiás, Faculdade de Enfermagem, Goiânia, GO, Brasil.

**Keywords:** Medication Errors, Safety Management, Nursing, Patient Participation, Quality of Health Care, Patient Safety, Errores de Medicación, Administración de la Seguridad, Enfermería, Participación del Paciente, Calidad de la Atención de Salud, Seguridad del Paciente, Erros de Medicação, Gestão da Segurança, Enfermagem, Participação do Paciente, Qualidade da Assistência à Saúde, Segurança do Paciente

## Abstract

**Objective:**

to analyze the perception of patients about health professionals’ adherence to safety barriers in medication administration.

**Method:**

cross-sectional and correlational study carried out in a hospital in the countryside of São Paulo, with a total of 249 adult patients admitted to the medical clinic. An electronic form developed by the researcher was used. Quantitative variables were analyzed in mean, median and standard deviation. Likert-type variables were calculated according to the perception score and the Bayesian Information criterion was used. The cutoff point for positive assessment of the patients’ perception was 0.75.

**Results:**

the average perception score was 0.29 and, of the 15 barriers analyzed, eight are never adhered by professionals, in the opinion of most patients. Also, age was the only variable with statistical significance.

**Conclusion:**

the younger the patient, the better their perception of health professionals’ adherence to safety barriers in medication administration.

## Abstract (es)

**Objetivo:**

analizar la percepción de pacientes sobre la adhesión de los profesionales de salud a las barreras de seguridad en la administración de medicamentos.

**Método:**

estudio transversal y correlacional realizado en un hospital del interior de São Paulo, con 249 pacientes adultos hospitalizados en la clínica médica. Se utilizó un formulario electrónico desarrollado por la investigadora. Las variables cuantitativas se analizaron en términos de media, mediana y desviación estándar. Las variables tipo Likert se calcularon según el puntaje de percepción y se utilizó el criterio de Información Bayesiana. El punto de corte para la evaluación positiva de la percepción del paciente fue 0,75.

**Resultados:**

el puntaje de percepción promedio fue de 0,29 y, de las 15 barreras analizadas, ocho nunca son adheridas por los profesionales, en la opinión de la mayoría de los pacientes. Además, la edad fue la única variable con significación estadística.

**Conclusión:**

cuanto más joven es el paciente, mejor es su percepción de la adhesión de los profesionales de salud a las barreras de seguridad en la administración de medicamentos.

## Abstract (pt)

**Objetivo:**

analisar a percepção de pacientes sobre a adesão dos profissionais de saúde às barreiras de segurança na administração de medicamentos.

**Método:**

estudo transversal e correlacional, realizado em um hospital do interior de São Paulo, com 249 pacientes adultos internados na clínica médica. Utilizou-se um formulário eletrônico desenvolvido pela pesquisadora. As variáveis quantitativas foram analisadas em termos de média, mediana e desvio-padrão. As variáveis do tipo Likert foram calculadas de acordo com o escore de percepção, utilizando-se o critério de Informação Bayesiana. O ponto de corte à apreciação positiva da percepção do paciente foi 0,75.

**Resultados:**

a média do escore de percepção foi 0,29, sendo que, das 15 barreiras analisadas, oito nunca são aderidas pelos profissionais, na visão da maioria dos pacientes. Além disso, a idade foi a única variável com significância estatística.

**Conclusão:**

Quanto mais jovem o paciente, melhor a sua percepção sobre a adesão dos profissionais de saúde às barreiras de segurança na administração de medicamentos.

## Introduction

Medication errors are a major cause of care-related harm and death^(1)^. Worldwide, the costs for the treatment of these events correspond to approximately 1% of total health expenses^(2)^. Although they can occur at any stage of the medication use process^(2-3)^, administration errors are the most common^(2)^ and stand out as a challenge for professionals, patients and health institutions^(4)^.

Systematic reviews of the literature showed that one in five drug doses is administered incorrectly in inpatient units^(5)^ and that the most common errors were timing errors, dose errors, dilution errors, errors in infusion rate and omission^(6)^. The errors usually result in moderate or severe harm and affect mainly people with complex health or social needs, in addition to extremes of age^(1)^.

Extensive efforts were made to prevent administration errors, including changes in care processes and the implementation of new technologies^(4)^. Safety barriers are defined as a set of measures used by the health team to manage potential risks related to care^(7)^; therefore, they are important to ensure patient safety. However, researches revealed low adherence of health professionals to these barriers^(8-11)^, contributing to the increased risk of harm^(9)^ and poor health outcomes.

Safety barriers in medication administration include computerized provider order entry, automated drug delivery systems, barcodes for drugs and patients, smart infusion pumps for administering intravenous drugs, compliance with the nine rights in drug administration, protocols focused on the management of high-alert medications and hand hygiene practices^(4,12-14)^. These barriers can also be classified into three major groups: optimization of medication systems; supporting professionals in managing interruptions and distractions and encouraging patient engagement in managing their own care^(15)^.

The safe administration of medications has been highlighted in patient safety. In a research carried out in an emergency service of a hospital in Minas Gerais, with the objective of analyzing the actions taken to improve the quality and the challenges of the nursing team to promote safe care in the administration of medication, a semi-structured interview was conducted with the professionals. The results revealed gaps in knowledge regarding the nine rights of drug administration, as well as inadequate staffing and lack of knowledge about new drugs. The researchers concluded that managers need to invest in training and in engaging patients in decision-making about health care^(16)^.

It is clear that the nursing team plays a major role in preventing errors in medication administration. Therefore, reducing potential risks at this stage of the medication process is essential to improve the quality of care^(17)^. Furthermore, the inclusion of patients in the process will allow their engagement in decision-making and in the search for information about care options^(18-19)^. For these reasons, patient participation in the prevention of administration errors should be encouraged.

Despite the exponential increase in attention to the participation of patients in care processes, and the various barriers implemented by health managers to reduce administration errors, the lack of research aimed at analyzing the patients’ perception of professional adherence is irrefutable to safety barriers^(20)^.

Considering the above, the aim of this study was to analyze the patients’ perception of health professionals’ adherence to safety barriers in medication administration.

In this study, perception was defined as the relationship established between one person and another, including an object and/or an event during the interrelationship. Thus, each individual presents their own perception of the relationship, of what is seen or identified by the other subject^(21)^.

## Method

### Type of study

This is a quantitative, cross-sectional and correlational study^(22)^. For its description, the STROBE guidelines (Strengthening the Reporting Observational Studies in Epidemiology) were used, which provide a formal and systematized structure for the criteria and methods for the selection of participants^(23)^.

### Study setting

The study was carried out in the medical clinic of a philanthropic hospital in the city of Franca, São Paulo, Brazil. The hospital has 206 beds and is a regional reference for urgency and emergency services in medium and high complexity. It has four Gold Quality certifications, including the hospital quality certification (HQC). The medical clinic was selected because it has a greater number of admissions/month and patients, in general, remain hospitalized for a longer period of time.

### Period of study

May 2019 to June 2020.

### Population

Adult patients admitted to the medical clinic unit of a philanthropic hospital in the city of Franca, São Paulo, Brazil.

### Selection criteria

Patients hospitalized for at least 2 days, capable of verbal communication and oriented in time, space and about the person. Patients in isolation during the period of data collection were not included in the study.

### Sample

The convenience sample consisted of a total of 249 patients hospitalized from June 2019 to September of the same year.

### Data collection instrument

An electronic form was created based on the literature on the subject^(2-15)^; it was divided into three parts and included the sociodemographic and clinical variables of the patients (gender, education, history of previous hospitalizations and time elapsed between the last and current hospitalization); conduct of health professionals in relation to medications used regularly at home; and safety barriers in drug administration. The questions related to the variables “behavior of health professionals in relation to medicines regularly used at home” and “safety barriers in medication administration” were made available on a Likert-type scale of five alternative answers (always, sometimes, never, I do not know, does not apply). The instrument was validated for face and content by a panel consisting of five experts. It was also submitted to a pilot study with 10 patients to verify its suitability, which legitimized its employability.

### Data collection

Structured interview was conducted in the ward, in the afternoon, lasting between 35 and 40 minutes. Patient privacy was maintained using screens. The responses were registered in the electronic form by the researchers, using a mobile device. Demographic and clinical data were obtained from the participants and the patients’ medical record.

Participants were approached by the researcher or by properly trained research assistants (three students from the 4^th^ year of the Undergraduate Nursing Course). The objectives were presented to the participants who, after voluntarily accepting to participate in the research, were asked to sign the Informed Consent Form.

### Data analysis

In data analysis, quantitative variables (gender, education and history of previous hospitalizations) were presented as absolute and relative frequencies, while continuous variables (patient age, how long was hospitalized and time since last hospitalization) were analyzed in terms of mean, median and standard deviation.

In the analysis of the Likert scale responses, the following scores were used for the alternatives: 1 (Always), 0.5 (Sometimes) and 0 (Never, I do not know and Does not apply - NA). The mean of the patients’ perception score was calculated, whose resulting value was in the range between zero and one [0-1]. Values greater than or equal to 0.75 were considered a positive perception of health professionals’ adherence to safety barriers in medication administration.

For the analysis of the standardized score, the Beta distribution (BE) or the inflated Beta distribution of Zeros and/or Ones (BEINF) was adopted, which belongs to the class of generalized additive models for position, scale and shape. As independent variables for the model, the following were analyzed: age (in years old), sex (male/female), education (no education/1 to 4 years/5 to 8 years/9 to 11 years/over 11 years), history of previous hospitalization (yes/no) and time between the last hospitalization and the current one (in years). The last one was only present for participants who answered “yes” to the item that dealt with a previous history of hospitalization.

Regarding the total score of patient perception, the selection of the distribution of the response variable was performed using the Bayesian Information (BIC) criterion. The model with the lowest BIC value was selected. To assess the adequacy of the response variable, the Shapiro-Wilk Normality test was applied on the adjustment residuals. Analyzes were performed using the R software version 3.6.1 and a significance level of 5% (α = 0.05) was considered.

### Ethical aspects

The study was approved by the Research Ethics Committee, via *Plataforma Brasil* (CAAE No. 11945618 2 3001 5438), according to Resolution 466/2012 of the National Health Council^(24)^.

## Results

Of the 249 (100%) patients, most were men (127; 51.0%), with 5 to 8 years of education (90; 36.1%) and a history of previous hospitalizations (230; 92.4%). The mean length of stay was 8.05 days (5.00 ± 9.60) and the mean time between the last and current hospitalization was 5.97 years (2.00 ± 7.66).

As for information about safety in drug administration, most patients (227; 91.2%) said they had not received it in their last hospitalization. As for the conduct of health professionals related to medications in continuous use at home, 65 (26.1%) patients said they had been instructed not to interrupt their use during the hospitalization period. However, 129 (51.8%) were not warned about the importance of keeping medications at home.

Table 1 shows the perception of patients about the adherence of health professionals to safety barriers in medication administration, indicating that, of the 15 barriers analyzed, eight (61.5%) are never adhered to by the professionals, in the perception of most of the patients. Also, more than 80% of patients said that professionals never report on the importance of drug allergy.

Regarding the identification bracelet, 83.8% (n = 207) of the patients stated that professionals never use at least two identifiers to confirm the right patient before administering the medication. Regarding hand hygiene, 65 (26.1%) patients stated that nursing professionals “never” perform the procedure before administering the medications.

**Table 1:** Distribution of patient responses about health professionals’ adherence to safety barriers in medication administration (N=249). Franca, SP, Brazil, 2019

SAFETY BARRIERS IN DRUG ADMINISTRATION	ANSWERS											
	ALWAYS		SOMETIMES		NEVER		I DO NOT KNOW		DID NOT ANSWER		DOES NOT APPLY	
	n	%	n	%	n	%	n	%	n	%	n	%
Professionals clean their hands with soap and water and/or hand sanitizer before administering the drug	88	35.3	69	27.7	65	26.1	24	9.6	3	1.2	-	-
I am informed about the importance of the ID bracelet	19	7.6	4	1.6	221	88.8	2	0.8	3	1.2	-	-
I am informed about the importance of the bed identification panel	9	3.6	4	1.6	231	92.8	2	0.8	3	1.2	-	-
My full name is checked before the medication	25	10.0	22	8.8	199	79.9	-	-	3	1.2	-	-
My ID bracelet is checked before administering the drug	17	6.8	34	13.6	185	74.3	10	4.0	3	1.2	-	-
At least two identifiers are used to confirm that I am the right patient, before the medication	11	4.4	28	11.3	207	83.8	-	-	3	1.2	-	-
I am oriented about the medications in use	85	34.1	48	19.3	111	44.6	2	0.8	3	1.2	-	-
I am informed about the dosage of medications administered during my stay in this hospital	45	18.1	24	9.6	175	70.3	2	0.8	3	1.2	-	-
I am informed about the action/function of the medication in use in this hospital	69	27.7	56	22.5	120	48.2	1	0.4	3	1.2	-	-
I am informed on the drug administration times used in this hospital	41	16.5	26	10.4	175	70.3	4	1.6	3	1.2	-	-
Information about the medications used in this hospital is easily understood	47	18.9	66	26.5	132	53.0	1	0.4	3	1.2	-	-
I was asked about allergies	36	14.5	11	4.4	83	33.3	-	-	3	1.2	116	46.6
I am informed on the time of infusion/administration of serums	26	10.4	9	3.6	103	41.4	-	-	3	1.2	108	43.4
I am informed on the importance of respecting the time of infusion/administration of serums	27	10.8	8	3.21	103	41.4	-	-	3	1.2	108	43.4
Professionals wear procedural gloves when administering injectable (intravenous) medications	203	81.5	14	5.6	28	11.2	-	-	3	1.2	1	0.4

Patients presented a negative perception of health professionals’ adherence to safety barriers in medication administration, regardless of gender, as the mean was 0.29 (0.25 ± 0.21).

**Figure 1:**
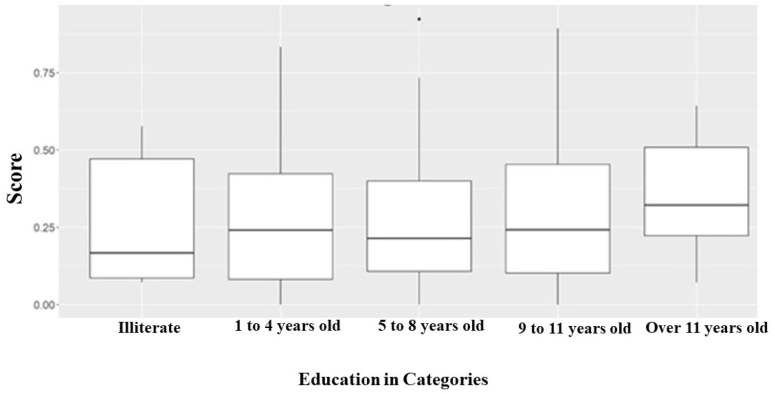
Bloxplot of the patients’ perception score on health professionals’ adherence to safety barriers in medication administration, according to educational level. Franca, SP, Brazil, 2019

Regarding education, 15% of illiterate patients had low or no perception of health professionals’ adherence to safety barriers in medication administration. Patients with 1 to 4 years or 9 to 11 years of education had an overall score equal to 0.25. Furthermore, patients with more than 11 years of education had higher scores when compared to the others. However, the perception was also negative, as it did not reach 0.75 (Figure 1).

Considering that the variable related to the time elapsed between the last hospitalization and the current one depended on the history of previous hospitalizations (only “Yes” answers), it was decided to carry out two adjustments of the inflated beta models. In the first model, the explanatory variables were used: age, gender, education and history of previous hospitalization. Only the age variable was statistically significant (Table 2).

**Table 2:** First adjustment of the inflated beta regression model for the explanatory variables: gender, previous hospitalization history, age and education. Franca, SP, Brazil, 2019

Explanatory variables	Estimate	**S.D.** *	t-value	Pr(>|t|)
(Intercept)	-0.1770	0.4385	-0.4037	0.6868
Male gender	-0.0009	0.1171	-0.0079	0.9937
History of previous admissions (Yes)	-0.1193	0.2265	-0.5266	0.5990
Age	-0.0088	0.0038	-2.3209	**0.0212**
1 to 4 years of education	-0.0260	0.2859	-0.0911	0.9275
5 to 8 years of education	-0.0779	0.2955	-0.2637	0.7923
9 to 11 years of education	-0.0211	0.3264	-0.0647	0.9484
˃ 11 years of education	0.1153	0.3316	0.3478	0.7283
**Dispersal**	**Estimate**	**S.D.** *	**t-valor**	**Pr(>|t|)**
(Intercept)	1.5272	0.0863	17.6904	**0.0000**
**Null**	**Estimate**	**S.D.** *	**t-valor**	**Pr(>|t|)**
(Intercept)	-3.0574	0.3085	-9.9101	**0.0000**

Note: Bold indicates the variables that showed statistical significance.

* SD = Standard Deviation

As noted in Table 2, for each increase of one year of life, a reduction of 0.87% is expected ([exp(-0.0088) - 1]*100) in the mean of the estimated perception score of 0-1, considering the other fixed variables (at the same level). The estimated dispersion was 4.6% (1.5272) and a probability of null perception of 4.5%.

In the second model, the explanatory variables were used: age, gender, education and time elapsed between the last hospitalization and the current one. Table 3 presents the result of the adjustment.

**Table 3:** Second adjustment of the inflated beta regression model for the explanatory variables: age, gender, education and time elapsed between the last hospitalization and the current one. Franca, SP, Brazil, 2019

Explanatory variables	Estimate	**S.D.** *	t-value	Pr(>|t|)
(Intercept)	-0.3595	0.4014	-0.8956	0.3715
Male gender	-0.0345	0.1190	-0.2900	0.7721
Age	-0.0093	0.0039	-2.3949	**0.0175**
1 to 4 years of education	0.0211	0.2859	0.0739	0.9411
5 to 8 years of education	-0.0374	0.2956	-0.1264	0.8995
9 to 11 years of education	-0.0319	0.3311	-0.0962	0.9234
˃ 11 years of education	0.0343	0.3376	0.1015	0.9193
Time between the last hospitalization and the current one	0.0146	0.0076	1.9206	0.0561
**Dispersal**	**Estimate**	**S.D.** *	**t-value**	**Pr(>|t|)**
(Intercept)	1.5580	0.0897	17.3695	0.0000
**Null**	**Estimate**	**S.Db.** *	**t-value**	**Pr(>|t|)**
(Intercept)	-2.9866	0.3090	-9.6646	0.0000

Note: Bold indicates the variables that showed statistical significance.

* SD = Standard Deviation

Again, only the age variable was statistically significant. It is verified that for each increase of one year of life, a reduction of 0.93% ([exp(-0.0093)-1]*100) in the average perception score (0-1) is expected when considering the other fixed variables (at the same level). The estimated dispersion was 4.7% (1.5580) and a probability of null perception of 4.8%.

The results showed that the younger the patient, the better their perception of the professionals’ adherence to safety barriers in medication administration.

## Discussion

The results showed a negative perception of patients about health professionals’ adherence to safety barriers in medication administration (mean score = 0.29). Still, patients with more than 11 years of education had a higher mean of perception in relation to the others. However, the total score was below 0.75.

Health literacy is a variable that is related to the patients’ knowledge and attitude in relation to the management of their own care^(25-26)^. Studies carried out in Japan^(27)^ and Germany^(28)^ revealed that adults who went through the experience of patient- and family-centered communication, as well as involvement in decision-making, were more likely to be satisfied with the care received, compared to those who had negative experiences in interpersonal communication and shared decision making. They concluded that greater efforts are needed to personalize care for people with low literacy^(27-28)^. In Brazil, there was a lack of evidence on this phenomenon. However, the importance of the partnership relationship with patients and families is recognized for the improvement of health outcomes, as well as for the promotion of a safer and more productive care environment for both parties^(29)^.

Of the 249 patients, 91% said they had not received information on error prevention strategies in medication administration. This is an alarming result, as patients are considered the last barrier to the prevention of administration errors^(15)^. Furthermore, researchers have shown that patients are often unaware of the medications prescribed during hospitalization, a fact that prevents them from becoming more actively involved in care planning^(30)^.

Honest, transparent and effective communication is an important barrier to the prevention of errors^(31)^, in addition to improving the experience in the patients’ journey. In this context, nurses play a vital role in communicating the care provided because they are endowed with vast knowledge and clinical experience that allows individualized care and focused on results.

Of the 15 barriers analyzed, most of them “never” (86.7%) were adhered by health professionals, in the patients’ opinion. Similar results were verified in a research carried out in a public hospital in Minas Gerais^(9)^. Of the 334 monitored doses, professionals did not adhere to good practices in 100%, including: patient identification through bracelet and bed (26.9%), information on the action and purpose of the medication (41.9%), identification of the drug (16.2%), disinfection of the connection (36.2%) and verification of the puncture device (14.4%).

On the other hand, in a study carried out in a hospital in the Midwest region of Brazil, most participants stated that the team frequently confirms the patients’ name, comprehensively explains the procedures and provides guidance on possible complications. According to the researchers, these actions contributed to patient satisfaction and were recommended for the promotion of safe care^(32)^.

Non-adherence to safety barriers can be understood as a risky behavior by the professional, which contributes to the occurrence of adverse events. However, these behaviors are often related to existing problems in the system and the complexity of health services^(33)^. In addition, the safety strategies published in the literature focus mainly on preventing errors based on human and system factors^(34)^. Although these approaches are important to reduce the impact of adverse events on health outcomes, research has shown the value of including the patient as an integral member of the team in error prevention strategies^(34-35)^.

In this study, 26.1% of patients reported that nursing professionals do not clean their hands and, for 11.2%, the team does not use gloves for administering injectable drugs. These results point to weaknesses in the processes that can negatively impact patients and workers.

A research carried out in a hemodialysis service in the country side of Sao Paulo^(36)^ showed that adherence to hand hygiene practices and the use of gloves are ideal. These practices are important barriers to reducing the transmission of infections in the context of health services, especially in times of pandemic, such as COVID-19. Furthermore, these measures are considered simple, of low cost and that have been proven to improve patient safety^(37)^. Continuing education programs are recommended to increase professionals’ awareness of the importance of these barriers and improve adherence to institutional protocols^(38)^.

With regard to allergies, 33.3% of the study participants stated that this information was never obtained by professionals. It is noteworthy that, in the investigated hospital, information about allergy is registered on a panel over the head of the bed, and the risk identification bracelet is not used. In a survey conducted at a university hospital in Spain with 283 hospitalized patients, with the objective of knowing the prevalence of drug allergy and the reactions presented by patients in the medical clinic, it was identified that 14.8% were allergic, of which 14.3% were related to medication and three to food. As a consequence, 33.2% required monitoring and in one case there was temporary harm. Furthermore, one third of the patients reported being allergic and that the information was registered in the medical record^(39)^.

Questioning the patient about allergies and providing the correct and timely information to the entire healthcare team are important barriers to reducing risks. Proactive, system-based measures should be designed and implemented to improve the drug administration process in healthcare services.

About the use of the identification panel and bracelet, more than 80% of the participants stated that they had never been informed about the importance of these safety barriers. Furthermore, 79% stated that their names are not checked before administering medication. It is noteworthy that, at the study hospital, the identification bracelet contains information related to the patient, such as full name, registration number and date of birth. However, the hospital does not have a barcode reader. These results corroborate research carried out in an emergency department in the city of Sao Paulo. According to the authors, failures in drug labeling and patient identification, before drug administration, were the most common errors and represented 47.9% and 62.3%, respectively^(40)^.

Complex, dynamic, busy and under-resourced health systems are a fertile ground for serious problems and the incorrect identification of patients is one of these problems^(41)^. Identification failures are responsible for high rates of avoidable harm^(32)^; therefore, nurses need to know and apply the main elements of patient identification, in order to ensure safety in the care provided, especially for patients who are unconscious and unable to respond for themselves.

The fact that most patients have never been informed about the dose and action of the drugs is worrying and reveals weaknesses in the safety culture of the investigated hospital. The result is similar to a research carried out in a public health institution in Parana. According to the researchers, 80.1% of the patients were unaware of the drug therapy used and 51.5%, the potential risks related to the use of medication^(42)^. Therefore, patients and family members must be informed about the benefits and risks related to the use of medications to improve treatment adherence and reduce potential errors^(2)^.

The results also showed that only age was statistically significant in the inflated beta regression analysis. Research carried out with the aim of evaluating this relationship is scarce. A better understanding of how and why age is associated with how the patient perceives interactions with health professionals can be useful for designing interventions and developing national policies that improve care delivery^(43)^, at all levels of care.

The patients’ involvement in their own safety is a strategy recommended by the WHO to improve health care^(44)^. Therefore, the education and teaching of the patient and family should be the nurses’ priority in care planning to favor the safe administration of medication.

Limitations of the study include the fact that data collection was performed using an electronic form developed specifically for the study. Therefore, comparisons between the results obtained and those of other national and international surveys were restricted. Furthermore, the perceptions of health professionals were not considered, and further research was recommended with the aim of evaluating the perception of teams and patients about barriers to preventing errors in administration and comparing the results with direct observation of the processes. The study was carried out in a hospital with a Gold Level Accreditation Certificate, so the results may not reflect those of institutions with other quality control seals.

## Conclusion

Age was the only variable with statistical significance, that is, the younger the patient, the better their perception of health professionals’ adherence to safety barriers in drug administration. The results may help health professionals and managers to improve the safety culture in hospitals, by determining patient and family engagement strategies in risk detection and planning actions aimed at preventing errors in medication administration.

## References

[1] Auraaen A, Slawomirski L, Klazinga N (2018). OECD health working papers: the economics of patient safety in primary
and ambulatory care. [Internet].

[2] World Health Organization (2019). Medication without harm: WHO’s third global patient safety challenge.
[Internet].

[3] World Health Organization (2016). Medication errors. Technical series on safer primary care. [Internet].

[4] PSNet (2019). Medication administration errors. [Internet].

[5] Keers RN, Williams SD, Cooke J, Ashcroft DM (2013). Prevalence and nature of medication administration errors in
health care settings: a systematic review of direct observational
evidence. Ann Pharmacother. [Internet].

[6] Moreira IN, Paes LAP, Araujo LM, Rocha FCV, Almeida CAPL, Carvalho CMS (2018). Erros na administração de medicamentos pela enfermagem: revisão
integrativa de literatura. BJSCR. [Internet].

[7] Rosen MA, DiazGranados D, Dietz AS, Benishek LE, Thompson D, Pronovost PJ (2018). Teamwork in healthcare: key discoveries enabling safer,
high-quality care. Am Psychol.

[8] Reeves S, Pelone F, Harrison R, Goldman J, Zwarenstein M (2017). Interprofessional collaboration to improve professional practice
and healthcare outcomes. Cochrane Database Syst Rev.

[9] Vória JO, Padula BLD, Abreu MNS, Correa AR, Rocha PK, Manzo BF (2020). Compliance to safety barriers in the medication administration
process in pediatrics. Texto Contexto Enferm.

[10] Mula CT, Solomon V, Muula AS (2019). The examination of nurses’ adherence to the ‘five rights’ of
antibiotic administration and factors influencing their practices: a mixed
methods case study at a tertiary hospital, Malawi. Malawi Med J.

[11] Lacerenza CN, Marlow SL, Tannenbaum SI, Salas E (2018). Team development interventions: evidence-based approaches for
improving teamwork. Am Psychol.

[12] Manias E, Kusljic S, Wu A (2020). Interventions to reduce medication errors in adult medical and
surgical settings: a systematic review. Ther Adv Drug Saf.

[13] Conselho Regional de Enfermagem de São Paulo (2017). Uso seguro de medicamentos: guia para preparo, administração e
monitoramento. [Internet].

[14] Ministério da Saúde (BR), Agência Nacional de Vigilância
Sanitária (2013). Protocolo de segurança na prescrição, uso e administração de
medicamentos. [Internet].

[15] Instituto para Práticas Seguras no Uso de Medicamentos (2019). Boletim ISMP Brasil. [Internet].

[16] Pinheiro TS, Mendonça ET, Siman AG, Carvalho CA, Zanelli FP, Amaro MOF (2020). Administração de medicamentos em um serviço de emergência: ações
realizadas e desafios para práticas seguras. [Internet]. Enferm Foco.

[17] Bessa D, Bueno E, Oliveira C, Elizabete R, Fonseca P, Mininel V (2019). Strategies to minimize medication errors in emergency units: an
integrative review. Rev Bras Enferm.

[18] World Health Organization (2016). Patient engagement: technical series on safer Primary Care.
[Internet].

[19] Schuh LX, Possuelo LG, Krug SBF (2019). Cultura de segurança do paciente em urgência e
emergência. RIP.

[20] Biasibetti C, Rodrigues FA, Hoffmann LM, Vieira LB, Gerhardt LM, Wegner W (2020). Segurança do paciente em pediatria: percepções da equipe
multiprofissional. REME.

[21] Vilela RPB, Jericó MC (2019). Implementing technologies to prevent medication errors at a
high-complexity hospital: analysis of cost and results. Einstein (São Paulo).

[22] Polit DF, Beck CT (2018). Fundamentos de pesquisa em enfermagem: avaliação de evidências para a
prática de enfermagem.

[23] Von Elm E, Altman DG, Egger M, Pocock SJ, Gøtzsche PC, Vandenbroucke JP, STROBE Initiative (2007). Strengthening the Reporting of Observational Studies in
Epidemiology (STROBE) statement: guidelines for reporting observational
studies. BMJ.

[24] Ministério da Saúde (BR), Conselho Nacional de Saúde (2013). Resolução nº 466, de 12 de dezembro de 2012. [Internet].

[25] Rodrigues FFL, Santos MA, Teixeira CRS, Gonela JT, Zanetti ML (2012). Relação entre conhecimento, atitude, escolaridade e tempo de
doença em indivíduos com diabetes mellitus. Acta Paul Enferm.

[26] Marques SRL, Escarce AG, Lemos SMA (2018). Letramento em saúde e autopercepção de saúde em adultos usuários
da atenção primária. CoDAS.

[27] Aoki T, Inoue M (2017). Association between health literacy and patient experience of
primary care attributes: A cross-sectional study in Japan. PLoS One.

[28] Altin SV, Stock S (2016). The impact of health literacy, patient-centered communication and
shared decision-making on patients’ satisfaction with care received in
German primary care practices. BMC Health Serv Res.

[29] Souliotis K, Agapidaki E, Peppou L, Tzavara C, Varvaras D, Buonomo O (2018). Assessing patient organization participation in health policy: a
comparative study in France and Italy. Int J Health Policy Manag.

[30] Garfield S, Jheeta S, Husson F, Lloyd J, Taylor A, Boucher C (2016). The role of hospital inpatients in supporting medication safety:
a qualitative study. PloS One.

[31] Pedro DRC, Silva GKTD, Dal Molin T, Oliveira JLCD, Nicola AL, Tonini NS (2016). Knowledge about patient hospital care received during your
admission. REME. [Internet].

[32] Arruda NLO, Bezerra A, Teixeira C, Silva AEBDC, Tobias GC (2017). Pacient perception of safety in health care provided by professionals in
a hospital emergency unit. Rev Enferm UFPE on line. [Internet].

[33] National Coordinator Council for Medication Error Reporting and
Prevention (2014). Reducing medication errors associated with at-risk behaviors by
healthcare professionals.

[34] McGinley P (2010). Patient engagement in patient safety: barriers and facilitators.
[Internet].

[35] Sharma AE, Rivadeneira NA, Barr-Walker J, Stern RJ, Johnson AK, Sarkar U (2018). Patient engagement in health care safety: an overview of
mixed-quality evidence. Health Aff (Millwood).

[36] Silva DM, Marques BM, Galhardi NM, Orlandi FS, Figueiredo RM (2018). Hands hygiene and the use of gloves by nursing team in
hemodialysis service. Rev Bras Enferm.

[37] World Health Organization (2009). WHO guidelines on hand hygiene in health care: first global patient
safety challenge clean care is safer care. [Internet].

[38] Carbajo ME, Cubero SJL, Lozano ASV, Del Pozo PE, Agulló GA, Colás SC (2020). Cross-sectional observational study of drug allergy in
hospitalized patients. Rev Rol Enferm [Internet].

[39] Mendes JR, Lopes MCBT, Vancini-Campanharo CR, Okuno MFP, Batista REA (2018). Types and frequency of errors in the preparation and
administration of drugs. Einstein (São Paulo).

[40] Llapa-Rodriguez EO, Silva LSL, Menezes MO, Oliveira JKA, Currie LM (2017). Safe patient assistance in the preparation and administration of
medications. Rev Gaúcha Enferm.

[41] Ferguson C, Hickman L, Macbean C, Jackson D (2019). The wicked problem of patient misidentification: How could the
technological revolution help address patient safety?. J Clin Nurs.

[42] Nieves CB, Díaz CC, Celdrán-Mañas M, Morales-Asencio JM, Hernández-Zambrano SM, Hueso-Montoro C (2017). Perception of ostomized patients about the health care
received. Rev. Latino-Am. Enfermagem.

[43] DeVoe JE, Wallace LS, Fryer GE (2009). Patient age influences perceptions about health care
communication. Fam Med. [Internet].

[44] World Health Organization (2013). Patient for patient safety: partnerships for safer health care.
[Internet].

